# The efficacy and safety of chemotherapy with or without anti‐PD‐1 for the first‐line treatment of advanced urothelial carcinoma

**DOI:** 10.1002/cam4.6671

**Published:** 2023-11-21

**Authors:** Fuxin Han, Zhaozhen Wu, Jiaxin Chen, Meicen Liu, Yi Hu

**Affiliations:** ^1^ Medical School of Chinese PLA Beijing China; ^2^ Department of Oncology The First Medical Centre Chinese PLA General Hospital Beijing China; ^3^ Beijing Chest Hospital Capital Medical University Beijing China; ^4^ Department of Genetic Engineering Beijing Institute of Biotechnology Beijing China; ^5^ School of Medicine Nankai University Tianjin China; ^6^ Department of Oncology The Fifth Medical Center, Chinese PLA General Hospital Beijing China

**Keywords:** adverse reactions, chemotherapy, efficacy, PD‐1 inhibitors, urothelial carcinoma

## Abstract

**Objective:**

To compare the efficacy and safety of first‐line anti‐PD‐1 combined with chemotherapy versus chemotherapy alone in patients with advanced urothelial carcinoma (UC).

**Method:**

Patients with advanced UC who received first‐line treatment of chemotherapy (*n* = 51, gemcitabine/paclitaxel [albumin‐bound] combined with platinum) or immunochemotherapy (*n* = 50, PD‐1 inhibitors plus chemotherapy) were enrolled. The efficacy and safety were analyzed between the two groups.

**Results:**

This study included data from 101 patients, including 51 patients in the chemotherapy group and 50 patients in the immunochemotherapy group. The median progression‐free survival of the immunochemotherapy group was significantly longer than that of the chemotherapy group (11.5 vs. 7.17 m, HR = 0.56, *p =* 0.009). The two groups' overall survival showed no significant difference (20.3 vs. 17.8 m, *p =* 0.204). The objective response rates and the disease control rates of the two groups were 38.0% versus 49.0% (*p =* 0.26) and 88.0% versus 80.4% (*p =* 0.29). The incidence of adverse reactions (AEs) in the immunochemotherapy group and chemotherapy group were 90.0% and 84.3% (*p =* 0.394), respectively, and the incidence of Grade III–IV AEs were 32.0% and 35.3% (*p* = 0.726), respectively.

**Conclusion:**

In the first‐line treatment of patients with advanced UC, anti‐PD‐1 therapy combined with chemotherapy might have better efficacy than chemotherapy alone, and AEs are similar between the two groups.

## 1 INTRODUCTION

Advanced urothelial cancer (UC) is a malignant disease with poor prognosis. According to Global Cancer Statistics 2020,^1^ bladder cancer is the 10th most prevalent cancer worldwide. Despite significant advances in diagnosis and treatment over the past 20 years, the 5‐year survival rate for patients with advanced UC remains at only 5%, the median overall survival (OS) is only approximately 18–19 months. Currently, the efficacy of available therapeutic regimens for these advanced patients remains limited,^2^ including standard cisplatin‐containing chemotherapy for the first‐line treatment.

Immune checkpoint inhibitors (ICIs) have been demonstrated to have great promise in treating a variety of malignant tumour, varying from non‐small‐cell lung cancer and malignant melanoma to renal cell carcinoma.^3^, ^4^, ^5^ Many studies have shown that ICIs have become a therapeutic choice for UCs. Since 2016, the Food and Drug Administration (FDA) has successively approved six ICIs (pembrolizumab, atezolizumab, nivolumab, avelumab, durvalumab and tislelizumab) as second‐line therapy for patients with advanced UC.^6^, ^7^, ^8^, ^9^, ^10^, ^11^, ^12^, ^13^ After that, in May 2017, accelerated approval for atezolizumab and pembrolizumab used in the first‐line treatment was granted to patients with locally advanced or metastatic UC who were ineligible for cisplatin‐containing chemotherapy by FDA.^14^, ^15^ Several studies reported that PD‐1/PD‐L1 inhibitors achieved some progress in treating patients in the early stages. The ABACUS study found that neoadjuvant use of pembrolizumab in early UC achieved a pathological complete response rate (CRR) of 31%,^16^ and the PURE‐01 study^17^, ^18^ also showed that pembrolizumab had good clinical efficacy for the neoadjuvant treatment of patients with operable UC. Besides, the KEYNOTE‐057 study reported a CRR of 41% among patients with BCG‐unresponsive bladder carcinoma in situ.^19^

Based on the efficacy data of atezolizumab in first‐line treatment for metastatic UC, the IMvigor130 study compared the efficacy of atezolizumab with the addition of chemotherapy versus atezolizumab versus chemotherapy in first‐line treatment for advanced UC.^20^ This study showed that atezolizumab‐based combined therapy significantly improved progression‐free survival (PFS) compared with chemotherapy (8.2 vs. 6.3 m) and showed encouraging interim OS data (16 vs. 13.4 m), supporting platinum‐based chemotherapy plus atezolizumab as a potential therapy for first‐line metastatic UC. However, there is still a lack of strong evidence for PD‐1/PD‐L1 inhibitors combined with chemotherapy as first‐line treatment for advanced UC patients with good general conditions. Therefore, in this study, we further explore the efficacy and safety of anti‐PD‐1 plus chemotherapy versus chemotherapy monotherapy by analyzing the data from 101 patients with advanced UC who received first‐line treatment in real‐world clinical practice.

## 2 METHOD

### 2.1 Study design and grouping

This study retrospectively collected data from patients treated with first‐line treatment for advanced UC between March 2017 and October 2021 in the PLA General Hospital. We screened eligible patients from the oncology department by retrieving electronic medical records, and the screening criteria were as follows: (1) histologically confirmed UC and disease stage IIIB~IV, according to TNM stage standards; (2) measurable disease and evaluable response; (3) ECOG ≤3; (4) received no less than two cycles of chemotherapy or PD‐1 inhibitor plus chemotherapy and (5) no severe cardiopulmonary disease, hereditary/acquired immunodeficiency disease or autoimmune disorder.

All eligible patients were divided into two groups: (1) chemotherapy group: patients who received gemcitabine/paclitaxel‐albumin ± cisplatin/carboplatin‐based chemotherapy; (2) immunochemotherapy group: patients treated with anti‐PD‐1 (pembrolizumab 200 mg q3w or nivolumab 3 mg/kg q2w or toripalimab 3 mg/kg q2w or sintilimab 200 mg q3w) and chemotherapy as described in the chemotherapy group.

### 2.2 Data collection and outcome evaluation

Two professional clinicians independently collected and reviewed clinicopathological data and treatment information, and two radiologists independently assessed all imaging materials according to Response Evaluation Criteria in Solid Tumors, version 1.1 (RECIST 1.1), and adverse events were assessed and recorded according to the National Cancer Institute Common Terminology Criteria for Adverse Events, version 5.0 (CTCAE 5.0). The cut‐off date was 30 September 2022.

The primary outcome endpoints were PFS (the time from treatment to disease progression or death from any cause) and OS (the time from initial treatment to death from any cause). The efficacy evaluation indicators also included the objective response rate (ORR) (the proportion of patients with CR/PR), disease control rate (DCR) (the proportion of patients with CR/PR/SD) and CRR (the proportion of patients with CR).

### 2.3 Statistical analysis

In this study, the sample size and power calculations were calculated by logistics regression based on the number of events needed to demonstrate efficacy for the coprimary endpoints of PFS. The Fisher's exact test or chi‐square test was used to compare the differences in clinical characteristics and responses between the two groups. For survival analysis, the Kaplan–Meier method was used to determine the median value and two‐sided 95% CIs with a *p* value determined by the log‐rank test. We set *p* < 0.05 as statistically significant. SPSS 22.0 software and R version 4.2.1 were used for statistical analysis.

### 2.4 Ethics

The study protocol was approved (S2020‐341) by the Chinese People's Liberation Army General Hospital ethics committee and complied with the principles of the Declaration of Helsinki and its contemporary amendments.

## 3 RESULTS

### 3.1 Patient characteristics

**FIGURE 1:**
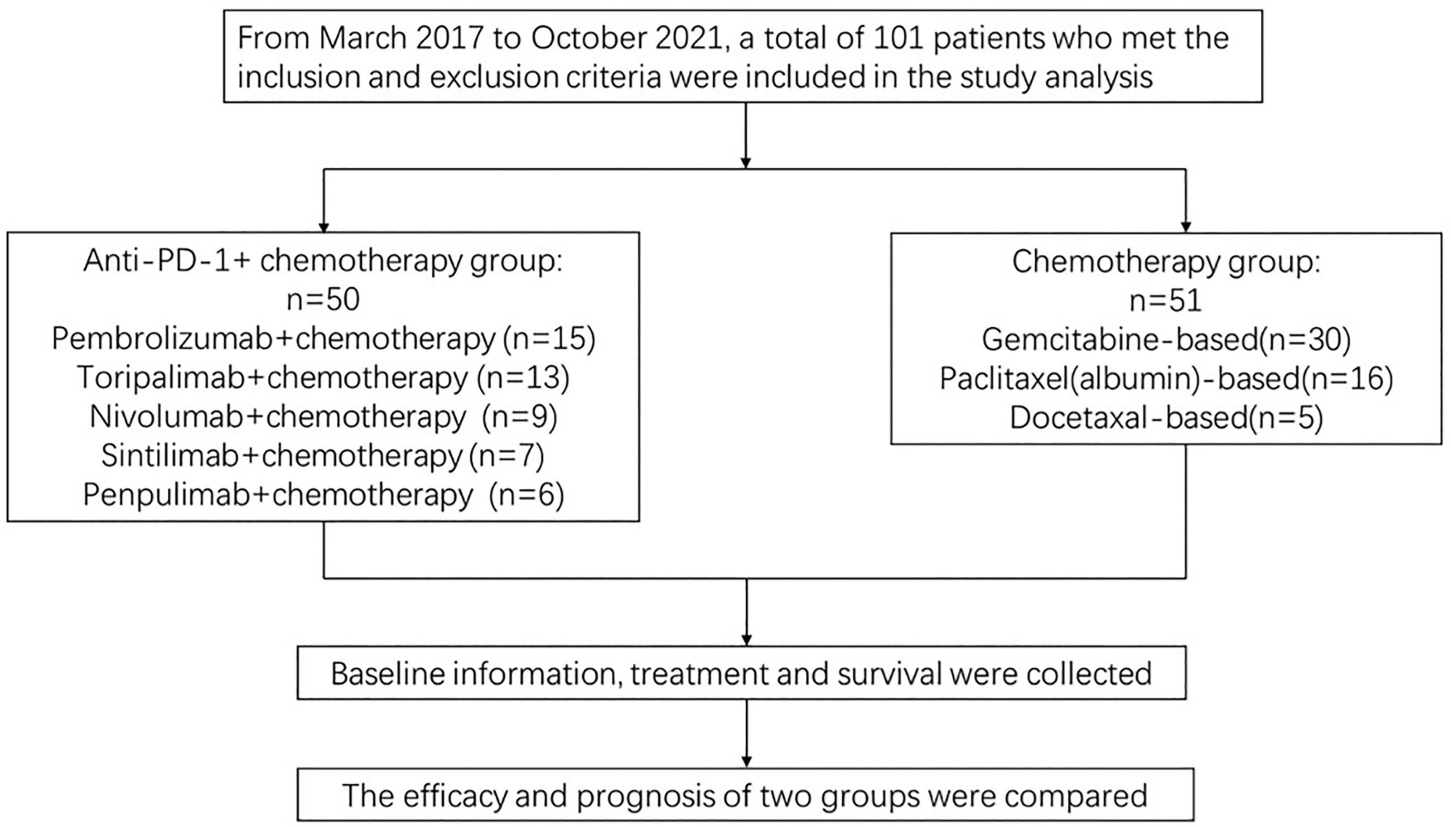
Flow diagram of the study.

In total, 101 eligible advanced UC patients were enrolled in our study, including 50 patients in the immunochemotherapy group and 51 patients in the chemotherapy group (Figure 1). We collected patient demographics and clinicopathological information, and these clinical characteristics were balanced between the two groups. The detailed descriptions are illustrated in Table 1.

**TABLE 1:** Baseline characteristics of patients in the two groups (*n*, %).

Characteristic	Chemotherapy (*n* = 51, %)	Immunochemotherapy (*n* = 50, %)	*p*‐Value
Gender			0.869
Male	37 (72.5)	37 (74.0)	
Female	14 (27.5)	13 (26.0)	
Age (years)			0.133
<65	34 (66.7)	26 (52.0)	
≥65	17 (33.3)	24 (48.0)	
ECOG score			0.199
0–1	40 (78.4)	44 (88.0)	
≥2	11 (21.6)	6 (12.0)	
Metastasis sites			
Lung	13 (25.5)	16 (32.0)	0.470
Liver	8 (15.6)	13 (26.0)	0.202
Bone	13 (25.5)	13 (26.0)	0.953
Differentiation			0.386
High grade	38 (74.5)	34 (68.0)	
Low grade	8 (15.7)	13 (26.0)	
Unknown	5 (9.8)	3 (6.0)	
Tumour site			0.356
Bladder	25 (49.0)	21 (42.0)	
Upper urinary tract	22 (43.1)	28 (56.0)	
≥2 sites	3 (5.9)	1 (2.0)	
Renal function			0.394
Normal	36 (70.0)	39 (78.0)	
Dysfunction	15 (29.4)	11 (22.0)	
Smoking history			0.485
Yes	24 (47.1)	27 (54.0)	
No	27 (52.9)	23 (46.0)	

### 3.2 Efficacy comparison between the two groups

**FIGURE 2:**
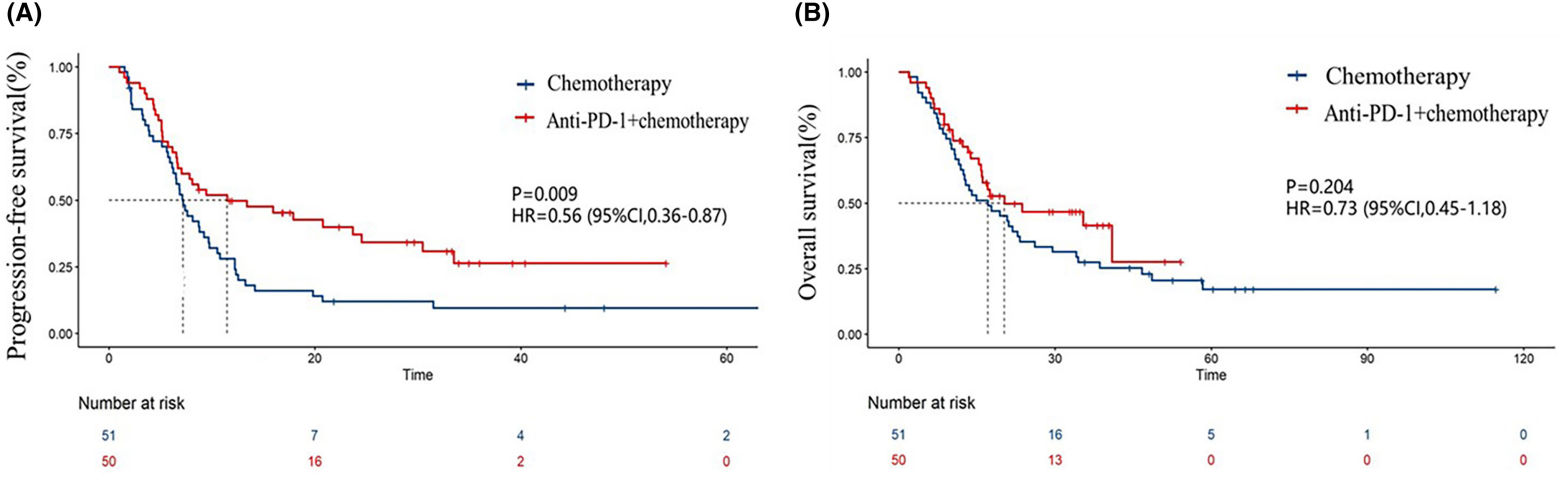
PFS curves (A) and OS curves (B) of patients in the two groups. PFS, progression‐free survival.

**FIGURE 3:**
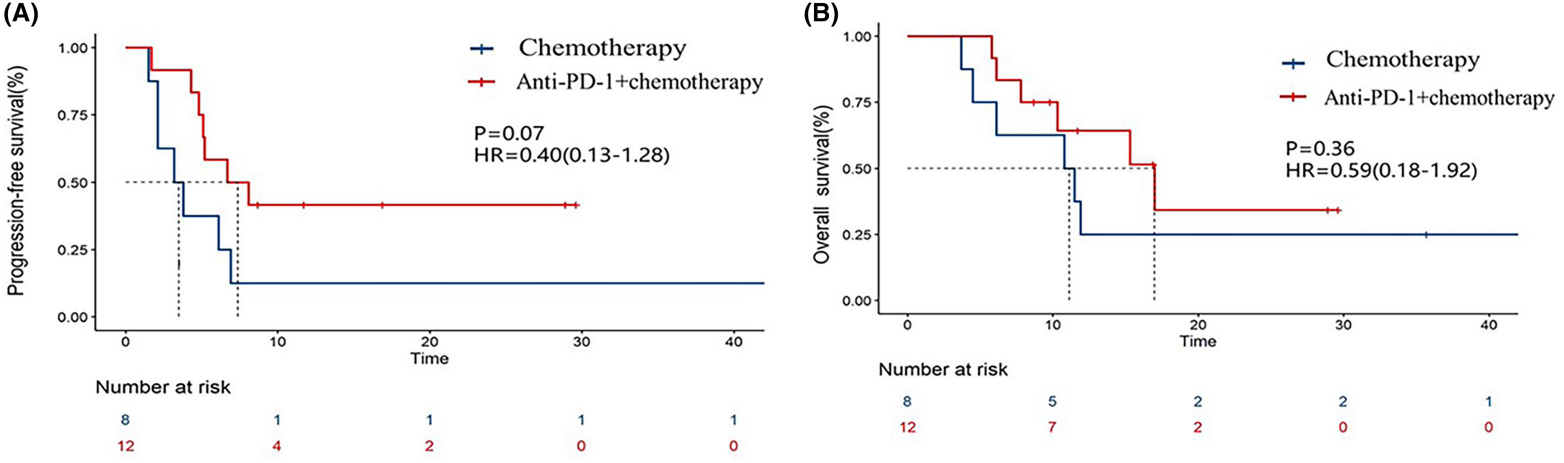
PFS curves (A) and OS curves (B) of patients with negative PD‐L1 expression in the two groups. PFS, progression‐free survival.

As of 30 September 2022, 78 (77.2%) PFS events and 67 (66.3%) deaths had occurred among all patients. The median PFS (mPFS) of the immunochemotherapy group was 11.5 months (95% CI, 1.26–21.67), which was significantly longer than the 7.2 months (95% CI, 5.94–8.4) of the chemotherapy group (HR = 0.56, *p =* 0.009; Figure 2A). The median OS (mOS) of the immunochemotherapy group was 2.5 months longer than that of the chemotherapy group (20.3 vs. 17.8 m), but there was no clear statistical difference (HR = 0.73, *p =* 0.204; Figure 2B). Through the forest map of the subgroup analysis, we observed a more obvious survival benefit (Figure S1). We also analyzed the influence of PD‐L1 expression on survival. For those with negative PD‐L1 expression, the mPFS (6.7 vs. 3.2 m, *p* = 0.070; Figure 3A) and mOS (17.0 vs. 10.8 m, *p* = 0.360; Figure 3B) of the combined treatment group were both higher than those of the chemotherapy group, although a significant difference was not achieved when comparing the mOS. In the combined treatment group, the phenomenon that patients with positive or negative PD‐L1 expression had similar PFS (11.5 vs. 6.7 m, *p* = 0.970; Figure S2A) and OS (20.3 vs. 17.0 m, *p* = 0.950; Figure S2B) is found by subgroup analysis based on PD‐L1 expression.

The ORRs of the combined treatment group and chemotherapy group were 38.0% (19/50) and 49.0% (25/51), respectively. Four patients achieved CR in the immunochemotherapy group and only one in the chemotherapy group. The DCRs of the two groups were 88.0% (44/50) and 80.4% (41/51), respectively. None of the data were statistically significant (Table 2).

**TABLE 2:** Clinical efficacy comparison between the two groups (*n*).

RECIST	Chemotherapy (*n* = 51)	Immunochemotherapy (*n* = 50)	*p‐*Value
CR	1	4	
PR	24	15	
SD	16	25	
PD	10	6	
ORR (%)	49.0	38.0	0.264
DCR (%)	80.4	88.0	0.295
CRR (%)	2.0	8.0	0.205

Abbreviations: CR, complete response; DCR, disease control rate; ORR, objective response rate; PD, progressive disease; PR, partial response; SD, stable disease.

### 3.3 Safety

The overall adverse events (AEs) were similar between the immunochemotherapy group and the chemotherapy group (Table 3). In these two groups, the overall incidence of AEs was 90.0% and 84.3%, and the incidence of Grade III–IV AEs in the two groups was 32.0% and 35.3%, respectively, no significant differences were found (P1 = 0.394, P2 = 0.726). The most common AEs included myelosuppression, gastrointestinal reaction, hepatotoxicity, fatigue and alopecia. No treatment‐related deaths occurred in any of the 101 patients.

**TABLE 3:** Comparison of adverse drug reactions between the two groups.

Adverse event	Grade I–IV			Grade III–IV		
	Chemotherapy (*n* = 51)	Immunochemotherapy (*n* = 50)	*p*‐Value	Chemotherapy (*n* = 51)	Immunochemotherapy (*n* = 50)	*p*‐Value
Any term	43 (84.3)	45 (90.0)	0.394	18 (35.3)	16 (32.0)	0.726
Leukopenia	23 (45.1)	20 (40.0)	0.604	8 (15.7)	5 (10.0)	0.394
Thrombocytopenia	9 (17.6)	9 (18.0)	0.963	6 (11.8)	4 (8.0)	0.527
Anemia	15 (29.4)	16 (32.0)	0.778	4 (7.8)	3 (6.0)	0.715
Gastrointestinal reaction	21 (41.2)	18 (36.0)	0.593	1 (2.0)	2 (4.0)	0.617
Hepatotoxicity	13 (25.5)	8 (16.0)	0.418	0	1 (2.0)	0.310
Fatigue	9 (17.6)	8 (16.0)	0.825	5 (9.8)	3 (6.0)	0.479
Alopecia	7 (13.7)	5 (10.0)	0.563	2 (3.9)	1 (2.0)	0.570
Skin rash	8 (15.6)	6 (12.0)	0.592	3 (5.9)	2 (4.0)	0.663
Oral mucositis	0	1 (2.0)	0.310	0	0	NA
Peripheral neurotoxicity	10 (19.6)	8 (16.0)	0.603	4 (7.8)	2 (4.0)	0.414
Blurred vision	0	1 (2.0)	0.617	0	0	NA
Fever	0	2 (4.0)	0.495	0	1 (2.0)	0.310
Pneumonia	0	1 (2.0)	0.310	0	0	NA

## 4 DISCUSSION

Recent work has highlighted the key role of PD‐1/PD‐L1 inhibitors in the treatment of different solid tumors.^21^ Studies have shown that ICIs might have stronger antitumor activity in cancers with higher mutation rates.^6^, ^22^ Data from The Cancer Genome Atlas demonstrated that UC ranked third in mutation rate and that a higher mutation rate might create a higher neoantigen load,^23^, ^24^ which may predict durable clinical benefit. Therefore, PD‐1/PD‐L1 monoclonal antibody may be promising in the treatment of UC. Clinical studies have confirmed their efficacy for the second‐line treatment of advanced UC and for the first‐line treatment of those ineligible for platinum‐based chemotherapy.^7^, ^8^, ^14^, ^15^ For those who are eligible for platinum‐based first‐line chemotherapy, however, whether the addition of immunotherapy can improve the survival of advanced UC is unclear.^20^, ^25^

On the basis of a standard chemotherapy regimen, it is worth exploring whether the combination of ICIs can bring more obvious benefits to patients with better tolerance, and there is still a lack of real‐world data on first‐line immunochemotherapy. Therefore, we observed and evaluated the real‐world efficacy of anti‐PD‐1 combined with chemotherapy in patients with advanced UC. In this retrospective study, we found that the ORR and DCR of the two groups were comparable; and the PFS and OS of the combined treatment group were longer than those of the chemotherapy group. Although the difference in OS was not statistically significant (*p =* 0.204), which might be due to a shortage of OS time, certain survival benefits were still observed in the immunochemotherapy group in terms of survival time and survival curve. Our results are generally consistent with previous studies^20^, ^25^, ^26^, ^27^, ^28^ and support PD‐1/PD‐L1 inhibitors plus platinum‐based chemotherapy as a promising treatment to improve efficacy. Clinical studies KEYNOTE‐361 and IMvigor130 both compared the efficacy of first‐line chemotherapy with immunochemotherapy in patients with advanced UC. Although the conclusions of these two studies were not completely identical, and the efficacy advantage of the combination therapy group was not as significant as in non‐small‐cell lung cancer, prolonged PFS and OS were observed, which was similar to the results of our study.

From the perspective of safety, the two groups showed similarities in adverse events and were mainly Grade I–II AEs. Compared with the chemotherapy group, the new AEs of the immunochemotherapy group included fever, pneumonia and blurred vision, which were thought to be related to immunotherapy, and these immune‐related AEs were often mild with a lower incidence. Consistent with the safety data of IMvigor130 and KEYNOTE‐361,^20^, ^25^ we found that compared with chemotherapy, the safety of PD‐1/PD‐L1 inhibitors combined with chemotherapy was similar and tolerable.

Results from IMvigor130, the most concerned Phase III clinical trial showed that atezolizumab combined with chemotherapy had encouraging median PFS (8.2 vs. 6.3 m) and OS (16 vs. 13.4 m) compared to chemotherapy alone.^20^ The median PFS and OS in our study were slightly longer than those in IMvigor130 and other clinical trials, possibly because our study was based on real‐world data in the Asian population, and patients may have received not only systemic therapy such as chemotherapy alone or immunochemotherapy but also local treatments such as radiotherapy and tumour‐reducing surgery to reduce tumour load. Despite inconsistencies due to differential approaches in study design, drugs and study methods, our results are consistent with IMvigor130 and several meta‐analyses, confirming that PD‐1 blockade combined with chemotherapy is promising for the first‐line treatment of advanced UC and that the safety is tolerated.

An ideal biomarker is still absent to sort the beneficial population of UC so far. The conclusion that PD‐L1 expression is entwined with the efficacy of PD‐1/PD‐L1 inhibitors has been reported by several studies, such as the IMvigor‐210 and NCT01693562 studies; however, the CheckMate 275 found that PD‐L1 expression could not predict tumour response.^6^, ^8^, ^12^ In our study, we performed subgroup analysis in the immunochemotherapy group according to PD‐L1 expression levels in tumours (CPS ≥ 10 or TPS ≥ 1% or IC ≥ 5% or TC ≥ 50% as PD‐L1 positive), and our results showed that the mPFS and mOS were similar between the positive and negative PD‐L1 expression subgroups. For patients with negative PD‐L1 expression, the addition of anti‐PD‐1 still improved PFS. Such results might have been due to different detection technologies and different cut‐off points. It remains challenging to categorize populations that may benefit from anti‐PD‐1/PD‐L1‐based immunotherapy, and it is worth exploring biomarkers using molecular biology and tumor immunology.

There are still some limitations of our study: (1) our case number was limited, and a real‐world study with enlarged sample size is needed; (2) we did not carry out subgroup analysis based on different chemotherapy drugs because the chemotherapy of our study was complex and (3) the OS was not mature because 48% of patients did not die by the follow‐up deadline. As the first real‐world study to investigate the efficacy and safety of anti‐PD‐1 therapy in combination with chemotherapy as a first‐line treatment in patients with advanced UC, despite certain limitations, it further validates the feasibility of this combination regimen and provides valuable clues for future prospective studies. More significantly, this study has the potential to provide better treatment options for appropriate patients with advanced UC to improve the prognosis.

## 5 CONCLUSION

For patients with untreated advanced UC, the current standard chemotherapy regimens offer limited survival benefits. However, our findings suggest that the addition of anti‐PD‐1 may prolong the PFS or even OS without more serious AEs. Therefore, with the patient's full knowledge and consent, we recommend well‐tolerated patients who are particularly likely to benefit from immunotherapy to receive combination therapy of anti‐PD‐1 plus chemotherapy or participate in similar clinical trials with a view to extending patient survival.

## AUTHOR CONTRIBUTIONS

**Fuxin Han:** Data curation (equal); software (equal); writing – original draft (equal). **Zhaozhen Wu:** Writing – original draft (equal); writing – review and editing (equal). **Jiaxin Chen:** Data curation (equal); software (equal); writing – review and editing (equal). **Meicen Liu:** Conceptualization (equal); resources (equal); validation (equal). **Yi Hu:** Conceptualization (equal); investigation (equal); project administration (equal); supervision (equal); validation (equal).

## FUNDING INFORMATION

The authors declare that no funds, grants or other support were received during the preparation of this manuscript.

## CONFLICT OF INTEREST STATEMENT

The authors declare that there is no conflict of interest. The authors have no relevant financial or non‐financial interests to disclose.

## ETHICS STATEMENT

This is an observational study. The Ethics Committee of Chinese People's Liberation Army General Hospital confirmed that no ethical approval or written informed consent was needed. All patient data accessed complied with relevant data protection and privacy regulations. In addition, this paper does not contain any individual personal data in any form.

## Supporting information


Figure S1.

## Data Availability

The original contributions presented in the study are included in the article/supplementary material. Further inquiries can be directed to the corresponding author.
