# Supplementary material for: The efficacy and safety of chemotherapy with or without anti‐PD‐1 for the first‐line treatment of advanced urothelial carcinoma
Source: Cancer Med. 2023 Nov 21;12(23):21129–37. doi: 10.1002/cam4.6671 (PMC10726829; doi:10.1002/cam4.6671)
Supplement: Supplementary file 1 — Figure S1. [file CAM4-12-21129-s001.pdf]

Supplementary Materials

Supplementary Table S1 PD-L1 status measured by CPS, TPS, TC or IC.

Anti-PD-1+Chemotherapy group	PD-L1 status	Chemotherapy group	PD-L1 status
patient1	(-)	patient1	TC(-),IC(1%)
patient2	CPS 2	patient2	(-)
patient3	(-)	patient3	(-)
patient4	TC(-), IC(3%)	patient4	TC(1%),IC < 1%
patient5	TPS < 1%	patient5	TPS < 1%,CPS 1
patient6	(-)	patient6	TC<1%
patient7	TC<1%, IC(-)	patient7	TPS<1%, CPS 1
patient8	(-)	patient8	TC<1%
patient9	CPS 3		
patient10	TC(10%)		
patient11	TC(3%)		
patient12	TC(2%)		
patient13	TC(-),IC(5%)		
patient14	TPS 10%		
patient15	TPS 5%		
patient16	TC(70%)		
patient17	TC(65%)		
patient18	CPS 30		
patient19	IC(10%), TC(2%)		
patient20	TC(60-70%)		
patient21	CPS 50		
patient22	CPS 10		
patient23	TPS 1%		

Supplementary Figure S1 Forest map of the subgroup analysis of PFS (a) and OS (b) in the anti-PD-1 plus chemotherapy group versus the chemotherapy group.

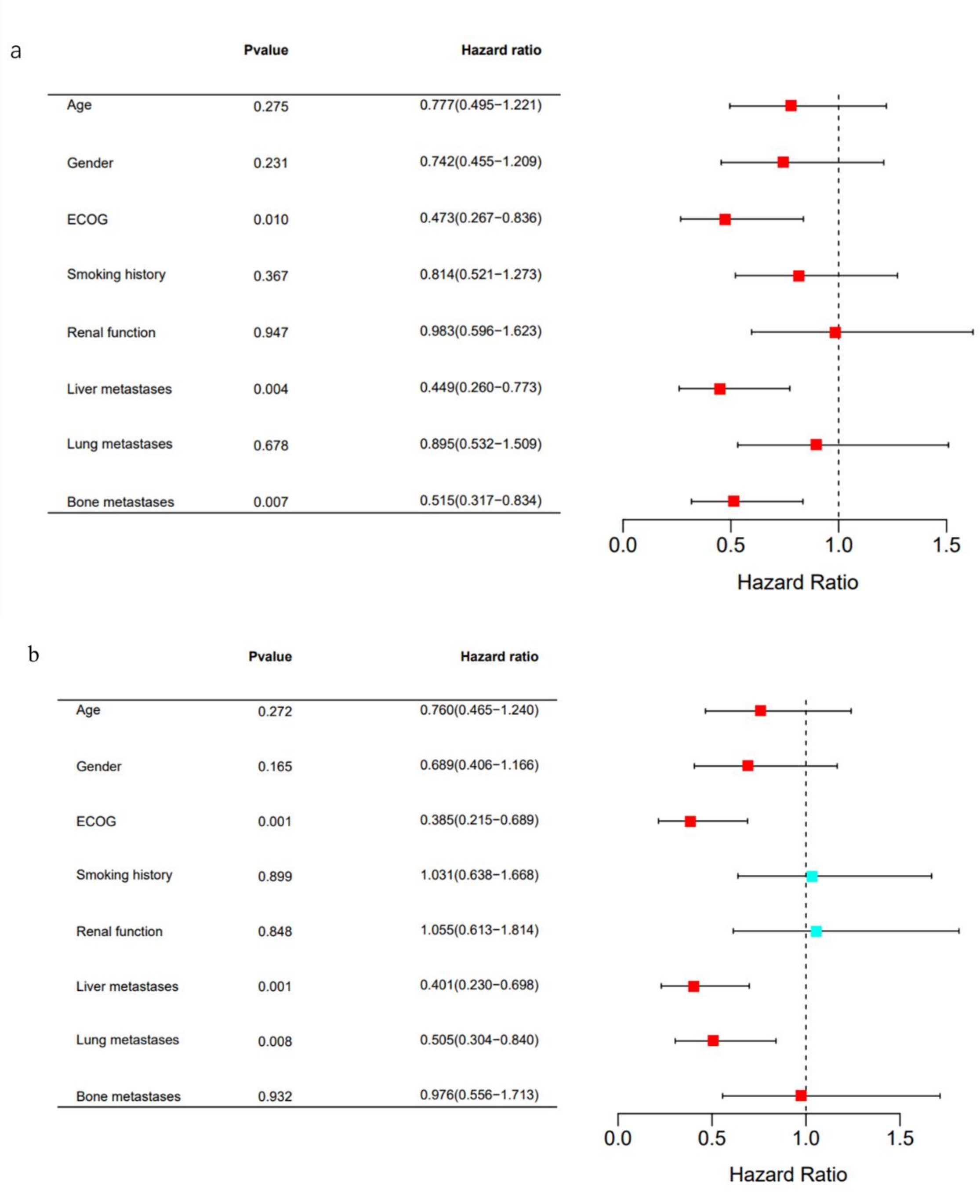

Supplementary Figure S2 Progression-free survival (a) and Overall survival (b) curves of patients with positive or negative PD-L1 expression in the anti-PD-1 plus chemotherapy group

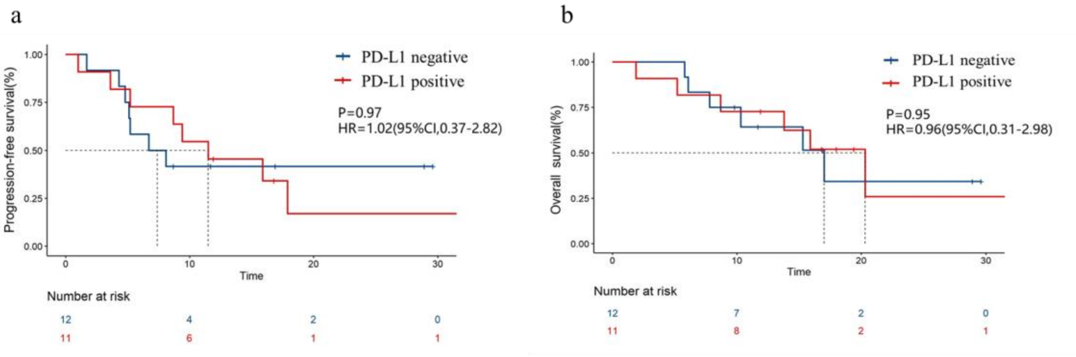
a
PD-L1 negative
PD-L1 positive
P=0.97
HR=1.02(95%CI,0.37-2.82)
Progression-free survival(%)
1.00
0.75
0.50
0.25
0.00
0
10
20
30
Time
Number at risk
12 4 2 0
11 6 1 1
b
PD-L1 negative
PD-L1 positive
P=0.95
HR=0.96(95%CI,0.31-2.98)
Overall survival(%)
1.00
0.75
0.50
0.25
0.00
0
10
20
30
Time
Number at risk
12 7 2 0
11 8 2 1
